# Piperine, a Natural Bioenhancer, Nullifies the Antidiabetic and Antioxidant Activities of Curcumin in Streptozotocin-Diabetic Rats

**DOI:** 10.1371/journal.pone.0113993

**Published:** 2014-12-03

**Authors:** Carlos Alberto Arcaro, Vânia Ortega Gutierres, Renata Pires Assis, Thais Fernanda Moreira, Paulo Inácio Costa, Amanda Martins Baviera, Iguatemy Lourenço Brunetti

**Affiliations:** Department of Clinical Analysis, School of Pharmaceutical Sciences, São Paulo State University - UNESP, Araraquara, São Paulo, Brazil; Stellenbosch University, South Africa

## Abstract

Knowing that curcumin has low bioavailability when administered orally, and that piperine has bioenhancer activity by inhibition of hepatic and intestinal biotransformation processes, the aim of this study was to investigate the antidiabetic and antioxidant activities of curcumin (90 mg/kg) and piperine (20 or 40 mg/kg), alone or co-administered, incorporated in yoghurt, in streptozotocin (STZ)-diabetic rats. The treatment for 45 days of STZ-diabetic rats with curcumin-enriched yoghurt improved all parameters altered in this experimental model of diabetes: the body weight was increased in association with the weight of skeletal muscles and white adipose tissues; the progressive increase in the glycemia levels was avoided, as well as in the glycosuria, urinary urea, dyslipidemia, and markers of liver (alanine and aspartate aminotransferases and alkaline phosphatase) and kidney (urinary protein) dysfunction; the hepatic oxidative stress was decreased, since the activities of the antioxidant enzymes superoxide dismutase, catalase and gluthatione peroxidase were increased, and the levels of malondialdehyde and protein carbonyl groups were reduced. The dose of 20 mg/kg piperine also showed antidiabetic and antioxidant activities. The treatment of STZ-diabetic rats with both curcumin and 20 mg/kg piperine in yoghurt did not change the antidiabetic and antioxidant activities of curcumin; notably, the treatment with both curcumin and 40 mg/kg piperine abrogated the beneficial effects of curcumin. In addition, the alanine aminotransferase levels were further increased in diabetic rats treated with curcumin and 40 mg/kg piperine in comparison with untreated diabetic rats. These findings support that the co-administration of curcumin with a bioenhancer did not bring any advantage to the curcumin effects, at least about the antidiabetic and antioxidant activities, which could be related to changes on its biotransformation.

## Introduction

Diabetes mellitus (DM) is a chronic endocrine disease characterized by disorders in the metabolism of carbohydrates, lipids and proteins due to a deficiency in the insulin production by pancreatic beta cells and/or to an increase in the insulin resistance in peripheral tissues. The International Diabetes Federation (IDF) estimated the world prevalence of DM for the years 2013 and 2035, showing alarming data: in 2013 more than 382 million people had diabetes, with estimation to rise to 592 million by 2035 [1]. These epidemic proportions of DM all around the world, in parallel with the rise of obesity, are explained by the combination of some factors, for example the rapid urbanization, nutritional transition, and diminished physical activity [2]. The metabolic impairments observed in DM, mainly those leading to hyperglycemia, account for the establishment of microvascular complications, such as nephropathy, neuropathy and retinopathy, diminishing the life quality and expectancy of diabetic individuals. In addition, DM is also associated with a high risk of macrovascular complications, including heart diseases and stroke, due to dyslipidemia, which is characterized by the “lipid triad”: elevated triglycerides (TG), low levels of high-density lipoprotein cholesterol (HDL-C), and the predominance of small dense low-density lipoprotein (sdLDL-C) particles [3].

Chronic hyperglycemia in DM accounts for the establishment of various impairments in many tissues, mainly those where the glucose uptake occurs in an insulin-independent manner; increased intracellular glucose levels results in an overproduction of reactive oxygen species (ROS) mostly via mitochondrial electron transport chain [4]. Moreover, the decreased activity of antioxidant enzymes, such as superoxide dismutase (SOD), catalase (CAT) and glutathione peroxidase (GSH-Px) has been also participating in the establishment of oxidative stress in DM [5]. Although it is known that ROS cause damage to biomolecules, these reactive species are also the initiator factors of various hyperglycemia-driven intracellular pathways, such as the polyol pathway, formation of advanced glycation end-products (AGEs), activation of protein kinase C (PKC) isoforms and increased flux through the hexosamine pathway; all these processes have a pivotal role in the development of long-term complications of DM [6]. In view of this, it is crescent the interest in the use of antioxidants, especially those from natural origin, in association with the conventional therapy for DM [7]–[9].

The presence of biologically active ingredients explains the potential of functional foods in preventing and/or reducing the risk of diseases. Plant sterols, fibers, alkaloids, and polyphenols are examples of compounds found in the functional foods those posses a wide spectrum of biological activities against human diseases [10], [11]. Curcumin is a polyphenol (diferuloylmethane), yellow pigment found in the rhizomes of turmeric (*Curcuma longa* L.) that has been traditionally used as food flavoring. In recent decades, curcumin has shown multifunctional pharmacological properties [12], including antidiabetic activity [13]. In addition, recent studies have postulated that some of the beneficial effects of curcumin against diabetes symptoms are related to its antioxidant potential [14], [15]. Study from our laboratory [16] showed that the treatment of streptozotocin (STZ)-diabetic rats with 90 mg/kg curcumin incorporated in yoghurt for 31 days improved various physiological and biochemical biomarkers classically altered in this experimental model of diabetes; the authors observed significant falls in the plasma levels of glucose, TG and aspartate and alanine aminotransferases, in the urinary levels of glucose, urea and protein, and an increase in the hepatic glycogen content. The treatment with curcumin-enriched yoghurt also decreased the food and water intake, urinary volume, and increased the body weight gain of STZ-diabetic rats. Also, the treatment of normal, non-diabetic rats with curcumin-enriched yoghurt did not promote any change in biomarkers for DM when compared with normal rats treated only with yoghurt or water.

The extensive range of pharmacological potentials of curcumin meets a problem related to its low bioavailability when administered orally [17], which could be limiting its therapeutic efficacy as well as the translation of experimental evidences into clinical trials, hindering the clinical approval of the curcumin use as a therapeutic antidiabetic agent [18]. The low bioavailability of curcumin is explained by its poor absorption, rapid rate of metabolism and excretion from the body [19]. Attempting to overcome this situation, several vehicles and associations with curcumin have been tested, such as curcumin-loaded nanoparticles, liposomes, micelles, among others [20], [21]. The oral co-administration of curcumin and bioenhancers of natural origin has been also investigated. Piperine, an alkaloid isolated from *Piper nigrum* fruits, is one of the first purified natural molecules with bioenhancer properties [22]. Piperine increases the bioavailability of drugs by inhibiting the biotransformation processes occurring in liver and intestine, due to its ability to inhibit the activity of various metabolizing enzymes, such as hepatic monooxygenases, aryl hydroxylases, N-demethylases, and UDP-glucuronyltransferases [22]–[24]. Piperine also inhibits cytochrome P450 3A4 (CYP3A4), a major enzyme participating in the first pass drug metabolism, and the P-glycoprotein transporter, which has been involved in the rapid efflux (systemic elimination) of drugs [25].

One of the first studies that investigated the piperine effects on the pharmacokinetics of curcumin in both human and rats was made by Shoba et al. [26]. The authors found that the co-administration of 2 g/kg curcumin and 20 mg/kg piperine to rats produced higher serum curcumin concentrations at 1 and 2 hours after the oral administration. The pharmacokinetic parameters of curcumin changed by piperine were: increased Tmax (time to reach the maximum serum concentration), decreased elimination half-life and total clearance elimination; overall, the curcumin bioavailability in rats was increased by 154%. In humans, the pharmacokinetic changes were more evident, since the bioavailability of curcumin was 2,000% increased 45 minutes after the co-administration with piperine. In another study, human subjects receiving for one week 2 g curcumin with 5 mg piperine also showed increased plasma curcumin levels (2-fold) in comparison with individuals receiving only curcumin, 2 hours after the last administration [17]. Increased half-life of orally administered curcumin was also found in rats receiving 500 mg/kg curcumin and 20 mg/kg piperine (28.88 hours) in comparison with curcumin alone (12.83 hours) [27]. Considering the above data, it is expected that the increased bioavailability of curcumin is a consequence of the piperine inhibition of biotransformation and/or efflux processes. In fact, many authors have suggested that the inhibition of glucuronidation process [17] and efflux transport [28] may be the mechanisms by which piperine increases the bioavailability of curcumin; however, to the best of our knowledge, a direct relationship between these piperine actions and the changes in the curcumin bioavailability was not yet described. In addition, knowing that both curcumin and piperine are inhibitors of cytochrome P, more research is needed to comprehend the biological consequences of the combined use of these phytotherapics [29].

Despite the studies showing the changes in the pharmacokinetic profile of curcumin when co-administered with piperine, little is known about the pharmacodynamic changes of curcumin in this association. Therefore, the aim of the present study was to investigate the changes promoted by the long-term treatment of STZ-diabetic rats with both curcumin and/or piperine incorporated in yoghurt on various biomarkers related to the metabolic and oxidative disturbances observed in this experimental model of DM.

## Materials and Methods

### Animals

Male Wistar rats weighing 140–160 g (6 weeks) were housed into individual metabolic cages, under environmentally controlled conditions of temperature (23±1°C) and humidity (55±5%) and with a 12 h light/dark cycle. Rats had free access to water and normal lab chow diet throughout the 45 days of experiment. The study was conducted in compliance with recommendations of the Brazilian College of Animal Experimentation (COBEA) and the experimental procedures received prior institutional approval by the Committee for Ethics in Animal Experimentation from the School of Pharmaceutical Sciences, UNESP, Araraquara, SP (resolution n° 08/2013).

### Induction of diabetes

After a initial period of adaptation, experimental diabetes was induced by a single intravenous injection of 40 mg/kg streptozotocin (STZ, Sigma Aldrich, USA) dissolved in 0.01 M citrate buffer (pH 4.5), in previously 14 h fasted rats. All animals were anesthetized with isoflurane. Three days after the STZ administration, rats with post-prandial glycemia values of approximately 25 mmol/L were used in the experiments. Plasma glucose levels were determined by the glucose oxidase method [30] using commercial kit (Labtest Diagnostica SA, Brazil).

### Experimental design and treatment

Curcumin (65%, Sigma Aldrich, USA), piperine (97%, Sigma Aldrich, USA) alone or in association were mixed with plain yoghurt (Nestlé, Brazil) with a homogenizer operating at 27,000 rpm for 90 seconds, at a controlled ambient temperature (25°C). Three days after diabetes induction, diabetic (D) rats were assigned into seven groups according to similar mean values of glycemia and body weight (10 rats/group): diabetic rats treated with yoghurt (DYOG); 90 mg/kg curcumin in yoghurt (DC90); piperine at doses of 20 mg/kg (DP20) or 40 mg/kg (DP40) in yoghurt; 90 mg/kg curcumin +20 mg/kg piperine (DC90P20); 90 mg/kg curcumin +40 mg/kg piperine (DC90P40) in yoghurt; 4U insulin (DINS). Rats of all groups (except DINS) were treated by gavage twice a day, at 08∶00 h and 17∶00 h, for 45 days. The curcumin, piperine and both were administered as a half dose in 0.5 mL of yoghurt, totaling 1.0 mL/rat/day of phytotherapics-enriched yoghurt. Rats of DINS group received two subcutaneous injections of insulin, 2U/rat each injection, at 08∶00 h and 17∶00 h, for 45 days.

During the experiment, several physiological and biochemical parameters were monitored. Body weight, food and water intake and urinary volume were monitored in a period of 24 h. An aliquot of urine was taken for the determination of the biochemical parameters glycosuria, urinary urea and proteinuria. Blood samples were collected from the tail in heparinized tubes (5,000 UI/mL) and plasma samples were obtained for the determination of glucose, triglycerides (TG), total-cholesterol (total-C) and HDL-cholesterol (HDL-C) and the activities of alanine aminotransferase (ALT), aspartate aminotransferase (AST) and alkaline phosphatase (ALP). The parameters body weight, water and food intake, urinary volume, glycemia, glycosuria, urinary urea and proteinuria were determined weekly. The plasma activities of AST, ALT and ALP and the plasma levels of TG, total-C and HDL-C were determined at every 15 days. The plasma levels of glucose, TG, total-C, HDL-C, ALT, AST and ALP and the urinary levels of protein and urea were determined using commercial kits (Labtest Diagnostica SA, Brazil). The o-toluidine method was used for the analysis of glycosuria [31].

At the end of the treatments, blood samples were collected from the tail for AST, ALT and ALP measurements and then the rats were euthanized by decapitation and blood was collected for the other biochemical analysis previously described. The *soleus* and *extensor digitorum longus* (EDL) skeletal muscles and the retroperitoneal and epididymal white adipose tissues were immediately removed and weighed, as was a piece of the liver, which was immediately stored at −80°C until analysis of oxidative stress parameters and activities of antioxidant enzymes.

### Oxidative stress biomarkers

#### Lipid peroxidation (LPO)

Liver samples (0.25 g) were homogenized in 1 mL of 1.15% potassium chloride at 4°C. The homogenates were centrifuged at 10,000 *g* for 10 min at 4°C and the supernatants were used for the analysis. Plasma and liver supernatants were previously deproteinized according Pilz et al. [32]. LPO dienes products, including malondialdehyde (MDA), were measured through thiobarbituric acid (TBA) reaction [33]. The MDA levels in liver were measured spectrophotometrically at 535 nm and the MDA levels in plasma were measured fluorometrically with excitation and emission wavelengths of 510 and 553 nm, respectively; 1,1,3,3-tetramethoxypropane was used as a standard. Results were expressed as µmol/L (plasma) or µmol/L/g tissue (liver).

#### Protein carbonyl groups (PCO)

The PCO levels were determined according to Levine et al. [34]; the carbonyl groups in proteins reacted with 2,4-dinitrophenylhydrazine (DNPH) to form 2,4-dinitrophenylhydrazone, which was estimated spectrophotometrically at 370 nm. Results were expressed as nmol/mg protein.

#### Reduced glutathione (GSH)

Non-protein sulphydryl groups represent an indirect measurement of GSH and were determined according to Sedlak and Lindsay [35], which measures the reduction of 5,5-dithiobis-(2-nitrobenzoic acid) (DTNB) at 412 nm. Results were expressed as mmol/L/g tissue.

### Measurement of the antioxidant enzymes activities

#### Sample preparation

Liver samples (0.1 g) were homogenized in 1 mL of sodium phosphate buffer (10 mmol/L, pH 7.4) at 4°C. The homogenates were centrifuged at 10,000 *g* for 10 min at 4°C and the supernatants were used for the analysis of SOD, CAT and GSH-Px activities. The protein levels in the supernatants were determined according to Lowry et al. [36], using bovine serum albumin as standard.

#### Superoxide dismutase (SOD) activity

SOD activity was evaluated according to Beauchamp and Fridovich [37], where xanthine and xanthine oxidase reaction generates superoxide anion (O_2_^•−^), which reduces nitroblue tetrazolium chloride (NBT) to a formazan product. The assay is based on the SOD inhibition of NBT reduction, monitored at 550 nm. Results were expressed as U/mg protein. One unit of SOD is defined as the enzyme amount required to inhibit the rate of NBT reduction by 50%.

#### Catalase (CAT) activity

CAT activity was measured according to Beers and Sizer [38] by monitoring the disappearance of hydrogen peroxide (H_2_O_2_) at 230 nm. Results were expressed as µKat/mg protein.

#### Glutathione peroxidase (GSH-Px) activity

GSH-Px activity was determined according to Rush and Sandiford [39]. GSH-Px catalyzes the oxidation of GSH in the presence of H_2_O_2_. In the presence of gluthatione reductase, the oxidized gluthatione is reduced to GSH with concomitant oxidation of NADPH to NADP^+^. NADPH disappearance was monitored at 340 nm. Results were expressed as µKat/mg protein.

### Statistical analysis

Data were expressed as mean ± standard error of mean (SEM). One-way analysis of variance (ANOVA) followed by Student-Newman-Keuls test was used to compare the temporal inter-group differences about the physiological, biochemical, oxidative stress parameters and the area under the curve (AUC). Paired Student’s t test was used to compare the intra-group changes in the physiological and biochemical parameters relative to day 0. Differences were considered significant at p<0.05. Statistical analyses were performed using the program Graphpad Instat 3.05 (GraphPad Software, USA).

## Results

### Plasma glucose levels

**Figure 1:**
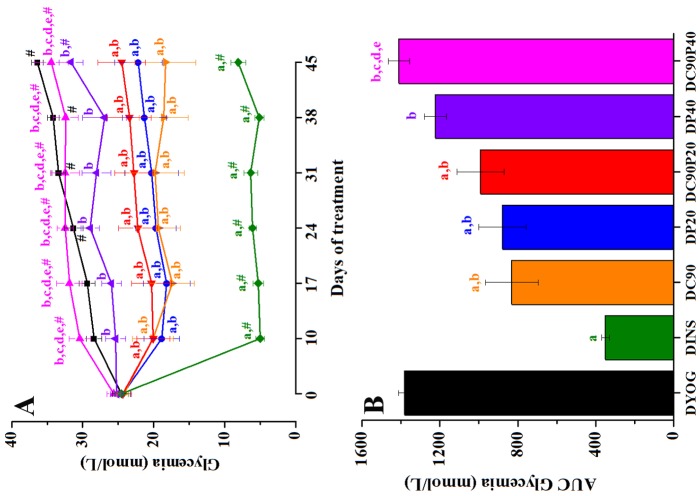
Glycemia (mmol/L) of STZ-diabetic rats treated with yoghurt enriched with curcumin, piperine, and curcumin+piperine. (A) Temporal responses, and (B) area under the curve (AUC). Values are expressed as means ± SEM, n = 10. Differences between groups were analyzed with One-way ANOVA followed by Student-Newman-Keuls test. a, differences with DYOG; b, differences with DINS; c, differences with DC90; d, differences with DP20; e, differences with DC90P20 (p<0.05). Differences in a same group relative to day 0 were analyzed with Paired Student’s t test. #, differences with day 0 (p<0.05).

According our results, it can be observed that the induction of diabetes in rats was effective, since all groups started the experiment with blood glucose levels of approximately 25 mmol/L. The DYOG group showed an increase in the glycemia levels throughout the experimental period, which represents a worsening of the diabetic state. The treatment of diabetic rats with insulin (DINS) was effective in reducing glycemia, maintaining the glucose levels near to 5.78 mmol/L, which is considered a value of normoglycemia to rats. In agreement with previous results of Gutierres et al. [16], the treatment of diabetic rats with curcumin-enriched yoghurt (DC90) improved the glycemia control, since it avoided the progressive increase in the glycemia as observed in DYOG. The treatment with both curcumin +20 mg/kg piperine (DC90P20) did not promote any beneficial effect on glycemia when compared with values of DC90 rats. However, when diabetic rats were treated with curcumin +40 mg/kg piperine (DC90P40), the improvement in the glycemia control promoted by curcumin was impaired, since the glycemia values were similar those observed in DYOG. The treatment with 20 mg/kg piperine (DP20) also prevented the progressive increase in the glycemia, while the values found in diabetic rats treated with 40 mg/kg piperine (DP40) were similar those observed in DYOG (Figure 1A). In the Figure 1B, it can be noted that the plasma glucose levels were decreased in DINS (75%), DC90 (40%), DP20 (37%), and DC90P20 (29%) groups when compared with DYOG. DP40 and DC90P40 groups did not have differences in glycemia levels in comparison with DYOG.

### Body weight

**Figure 2:**
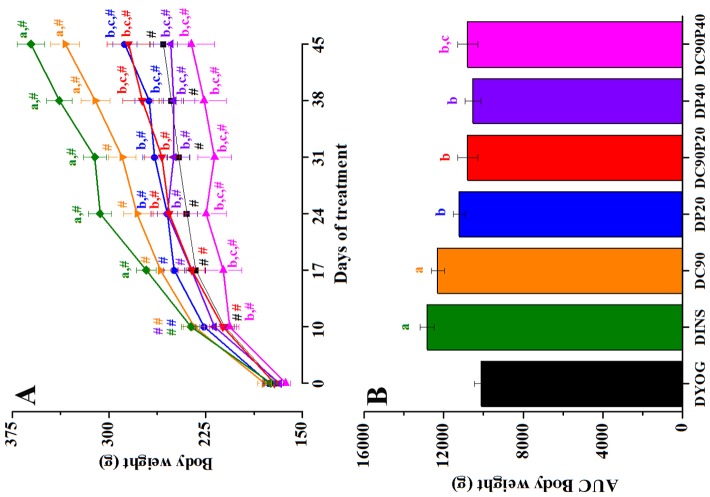
Body weight (g) of STZ-diabetic rats treated with yoghurt enriched with curcumin, piperine, and curcumin+piperine. (A) Temporal responses, and (B) area under the curve (AUC). Values are expressed as means ± SEM, n = 10. Differences between groups were analyzed with One-way ANOVA followed by Student-Newman-Keuls test. a, differences with DYOG; b, differences with DINS; c, differences with DC90 (p<0.05). Differences in a same group relative to day 0 were analyzed with Paired Student’s t test. #, differences with day 0 (p<0.05).

The lowest body weight gain observed in DYOG (Figure 2A) is corroborated by the minor weights of adipose tissues and skeletal muscles (Table 1). As expected, the DINS group recovered the ability to grow normally, from the 17^th^ day of treatment; DINS also showed the higher weights of adipose and muscle tissues. The DC90 group also showed an increased body weight gain, from 38^th^ day of treatment, corroborated by the increased weight of adipose tissues and EDL muscles. Both the DP20 and DP40 groups showed body weight values similar to DYOG, as well as a lower weight of adipose tissues and skeletal muscles. The DC90P20 group showed a body weight gain and the adipose and muscles weights similar of those observed with DYOG, showing that the benefit previously observed with curcumin alone was impaired in the presence of piperine. Similarly, both the body weight gain and the tissues weights of DC90P40 group were decreased in comparison with DC90, and had a trend to be lower than those of DYOG, at the end of the experiment. In the Figure 2B, it can be observed that the beneficial effect of curcumin on body weight was comparable to that of insulin, since the body weight was similar between DC90 and DINS groups and were 22% and 27% increased, respectively, in comparison with DYOG. Body weight values of diabetic rats treated with piperine (DP20 or DP40) or with both curcumin and piperine (DC90P20 and DC90P40) were similar those of DYOG.

**Table 1:** Weight of skeletal muscles (*soleus*, EDL) and white adipose tissues (epididymal, retroperitoneal) of STZ-diabetic rats treated for 45 days with yoghurt enriched with curcumin, piperine, and curcumin+piperine.

Parameters	DYOG	DINS	DC90	DP20	DC90P20	DP40	DC90P40
*Soleus* muscle (g)	0.123±0.007	0.163±0.005(a)	0.139±0.007(b)	0.116±0.006(b)	0.124±0.008(b)	0.113±0.008(b)	0.097±0.005(b, c)
EDL muscle (g)	0.109±0.008	0.168±0.005(a)	0.141±0.007(a, b)	0.109±0.008(b, c)	0.119±0.009(b, c)	0.094±0.009(b, c)	0.078±0.006(b, c, e)
Epididymaladipose tissue (g)	1.807±0.239	5.259±0.498(a)	3.386±0.480(a, b)	1.763±0.203(b, c)	2.512±0.442(b)	1.552±0.240(b, c)	1.212±0.083(b, c)
Retroperitonealadipose tissue (g)	1.038±0.229	3.807±0.565(a)	2.578±0.535(a, b)	0.703±0.158(b, c)	1.266±0.406(b, c)	0.685±0.210(b, c)	0.341±0.199(b, c)

Values are expressed as means ± SEM, n = 10, Differences between groups were analyzed with One-way ANOVA followed by Student-Newman-Keuls test. a, differences with DYOG; b, differences with DINS; c, differences with DC90; d, differences with DP20; e, differences with DC90P20 (p<0.05). Differences in a same group relative to day 0 were analyzed with Paired Student’s t test. #, differences with day 0 (p<0.05).

### Physiological parameters

In the Table 2 are shown the values of food and water intake and urinary volume of diabetic animals before (day 0) and after 45 days of the different treatments. After 45 days of experiment, classical symptoms for diabetes were observed in DYOG, such as polyphagia, polydipsia and polyuria, in comparison with day 0. As expected, the treatment of diabetic rats with insulin prevented all these symptoms. The treatment of diabetic rats with curcumin-enriched yoghurt also prevented the impairments in the physiological parameters related to insulin deficiency, since the food and water intake and the urinary volume in DC90 were similar between the day 0 and the day 45 and they were also decreased when compared with DYOG. Rats from DP20 and DP40 groups had minor food intake in comparison with DYOG, but the values of water intake and urinary volume were similar those of untreated diabetic rats. Food intake of diabetic rats treated with curcumin +20 mg/kg of piperine was similar that of DC90, and water intake and urinary volume were similar those of DP20. As observed for glycemia and body weight, the co-administration of curcumin and 40 mg/kg piperine seems to be detrimental to diabetes state, since impairments were found in food and water intake and urinary excretion in DC90P40 rats, in comparison with the effects of curcumin alone.

**Table 2:** Physiological parameters (food and water intake, urinary volume), lipid profile (TG, total-C, HDL-C) and urinary biomarkers (glycosuria, urinary urea, proteinuria) of STZ-diabetic rats before (day 0) and after (day 45) the treatment with yoghurt enriched with curcumin, piperine, and curcumin+piperine.

	Groups													
	DYOG		DINS		DC90		DP20		DC90P20		DP40		DC90P40	
Parameters	Day 0	Day 45	Day 0	Day 45	Day 0	Day 45	Day 0	Day 45	Day 0	Day 45	Day 0	Day 45	Day 0	Day 45
Foodintake(g/24 h)	28.14±1.45	46.06±1.53(#)	27.04±1.07	29.68±1.32(a)	27.25±1.21	33.06±2.89(a)	27.00±1.40	35.68±2.71(a,#)	28.93±1.52	33.89±2.64(a)	26.79±1.3	39.67±1.47(b,#)	27.57±0.66	43.79±1.40(b,#)
Waterintake(mL/24 h)	109.82±4.73	201.56±10.11(#)	88.87±11.52	30.29±3.58(a,#)	91.62±10.19	93.78±21.49(a, b)	100.25±8.25	186.53±16.14(b,#)	97.80±11.62	133.13±22.92(b)	111.67±9.64	190.38±10.67(b,#)	126.67±7.30	183.56±24.60(b,#)
Urinaryvolume(mL/24 h)	68.08±8.94	183.00±9.78(#)	56.53±6.86	27.77±6.32(a,#)	53.58±6.47	76.20±16.52(a, b)	65.33±8.70	134.00±15.24(b, c,#)	52.00±8.88	144.58±20.39(b, c,#)	63.86±8.81	137.57±13.69(b, c,#)	81.00±7.30	160.57±9.20(b, c,#)
TG(mmol/L)	1.04±0.10	3.33±0.51(#)	1.13±0.10	1.11±0.11(a)	1.07±0.14	1.36±0.14(a)	1.06±0.11	1.71±0.20(a,#)	1.14±0.09	1.82±0.18(a,#)	1.04±0.08	1.71±0.29(a,#)	1.19±0.13	2.37±0.44(b, c,#)
Total-C(mmol/L)	1.95±0.09	2.46±0.15(#)	1.95±0.09	1.48±0.09(a,#)	1.98±0.08	1.58±0.10(a,#)	1.96±0.08	1.88±0.09(a)	1.96±0.08	1.62±0.09(a)	1.95±0.07	1.83±0.25(a)	1.95±0.09	1.73±0.03(a)
HDL-C(mmol/L)	1.15±0.04	1.02±0.03	1.20±0.03	0.91±0.02	1.23±0.03	1.00±0.04	1.16±0.03	1.08±0.05	1.20±0.03	1.02±0.06	1.09±0.02	0.94±0.04	1.22±0.09	1.04±0.05
Glycosuria(mmol/L/24 h)	41.12±6.49	95.56±3.08(#)	32.50±2.43	5.36±2.57(a,#)	36.69±3.02	25.98±9.40(a)	35.72±6.32	59.19±12.24(a, b, c)	26.24±5.28	40.96±10.02(a, b)	32.63±5.19	69.27±6.32(b, c,#)	37.92±6.17	80.33±4.91(b, c.e.#)
Urinaryurea(mmol/L/24 h)	33.73±6.18	67.80±4.71(#)	31.19±1.80	24.10±3.32(a,#)	38.49±5.69	36.66±3.95(a)	28.04±2.83	40.59±8.30(a)	27.55±4.44	44.70±5.54(a, b)	36.06±4.01	50.70±3.23(a, b,#)	34.29±3.00	54.92±5.51(b, c, d, e, #)
Proteinuria(µmol/L/24 h)	13.02±0.99	28.45±4.03(#)	12.20±1.11	13.29±1.21(a)	11.34±1.27	16.92±2.30(a,#)	11.47±1.34	18.90±3.16(a,#)	11.89±1.34	17.59±1.79(a,#)	11.93±1.73	24.52±1.06(b,#)	11.88±1.34	31.81±3.32(b, c, d, e, #)

Values are expressed as means ± SEM, n = 10. Differences between groups were analyzed with One-way ANOVA followed by Student-Newman-Keuls test. a, differences with DYOG; b, differences with DINS; c, differences with DC90; d, differences with DP20; e, differences with DC90P20 (p<0.05). Differences in a same group relative to day 0 were analyzed with Paired Student’s t test. #, differences with day 0 (p<0.05).

### Lipid profile

The medium plasma levels of TG, total-C and HDL-C of all groups were very similar at day 0 (Table 2). At the end of the experiment, the DYOG group showed an impairment in lipid profile, with increased levels of TG and total-C, while the treatment with insulin avoided these disturbances, even decreasing the total-C levels in comparison with day 0. The treatment with curcumin, alone or co-administered with 20 mg/kg piperine, also improved TG, which levels were lower than DYOG; however, the final TG levels in DC90P20 group were increased in comparison with day 0. At the end of the treatment, the DP20 and DP40 groups showed a reduction in TG levels when compared with DYOG, but the values were increased in comparison with day 0. Here again, rats of DC90P40 group showed the same impairment in TG levels as observed in DYOG (Table 2). The levels of total-C were reduced in plasma of diabetic animals treated with curcumin alone (DC90) or with curcumin +20 mg/kg piperine (DC90P20), in comparison with values of day 0, in the same group, and in comparison with DYOG. Although not different in relation to time 0, the treatment of diabetic rats with piperine (DP20 or DP40) avoided the increase in the total-C levels as observed in DYOG. Total-C was the unique parameter that was improved with curcumin +40 mg/kg piperine treatment: after 45 days of experiment, rats of DC90P40 group showed total-C levels diminished when compared with DYOG (Table 2). Neither the chronicity of the experimental groups or the different treatments led to changes in the HDL-C levels (Table 2).

### Urinary biomarkers (glycosuria, urinary urea and proteinuria) and kidney function

At the beginning of the experiment, all groups showed similar values of glycosuria, urinary urea and proteinuria (Table 2). Increased levels of these urinary biomarkers were observed in DYOG after 45 days, as a consequence of uncontrolled glycemia levels (glycosuria), increased amino acid catabolism (urinary urea) and kidney dysfunction (proteinuria), typical symptoms of long-term diabetes. As expected, the treatment with insulin avoided the increase in these urinary biomarkers, even decreasing the glycosuria and the urinary urea levels in comparison with day 0, and prevented the establishment of kidney dysfunction. The glycemia control observed in diabetic rats treated with curcumin was reflected in the urinary biomarkers: the urinary levels of glucose, urea and protein in rats of DC90 and DC90P20 groups after 45 days of treatment were lower than values of DYOG; however, even reduced, the proteinuria values at the end of the experiment were higher than those of day 0, in both groups (Table 2). As observed to various other parameters previously described, the beneficial effects promoted by curcumin in urinary biomarkers were abrogated in the presence of 40 mg/kg piperine (DC90P40). The treatment with 20 mg/kg piperine (DP20) also improved the urinary biomarkers levels in diabetic rats in comparison with DYOG, but they were impaired in the treatment with 40 mg/kg piperine (DP40) (Table 2).

### Liver function profile

**Figure 3:**
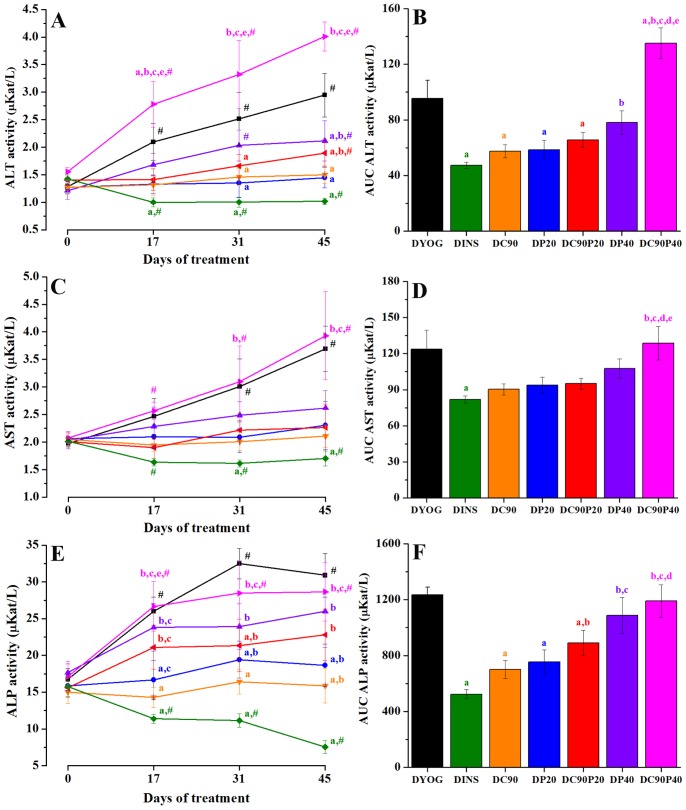
Plasma activities of ALT, AST, and ALP (µKat/L) of STZ-diabetic rats treated with yoghurt enriched with curcumin, piperine, and curcumin+piperine. (A) (C) and (E) Temporal responses, and (B) (D) and (F) area under the curve (AUC). Values are expressed as means ± SEM, n = 10. Differences between groups were analyzed with One-way ANOVA followed by Student-Newman-Keuls test. a, differences with DYOG; b, differences with DINS; c, differences with DC90; d, differences with DP20; e, differences with DC90P20 (p<0.05). Differences in a same group relative to day 0 were analyzed with Paired Student’s t test. #, differences with day 0 (p<0.05).

Changes in the liver function of STZ-diabetic rats due to the experimental diabetes and/or the different treatments were monitored by measuring the plasma levels of ALT, AST, and ALP. The levels of ALT, AST and ALP were progressively increased in plasma of DYOG rats. As expected, the treatment of diabetic rats with insulin (DINS) prevented this increase; these data are in agreement with previous results [40]. Maintenance in the ALT, AST and ALP levels close to values of day 0 was observed in diabetic rats treated with curcumin (DC90) or with 20 mg/kg piperine (DP20) (Figures 3A, 3C, 3E).

It is interesting to note that both ALT and ALP seem to be more sensitive biomarkers of liver dysfunction in comparison with AST. ALT levels were slightly increased in plasma of DC90P20 rats in comparison with the beginning of the experiment, as well as in DP40 rats, a profile not observed in DC90 or DP20 groups (Figure 3A). The activity of ALP was also increased in plasma of DC90P20 rats in comparison with DINS, but still remained similar to values of DC90 or DP20 groups (Figure 3F). In rats treated with 40 mg/kg piperine, the ALP levels were increased and did not differ from values found in DYOG (Figure 3F).

The levels of ALT, AST and ALP were markedly increased in plasma of diabetic rats treated with curcumin +40 mg/kg piperine; the levels of AST (Figures 3C and 3D) and ALP (Figures 3E and 3F) in DC90P40 rats were similar those found in DYOG. The development of liver dysfunction after the treatment with curcumin +40 mg/kg piperine was noted by the ALT levels, since the activity of this enzyme in DC90P40 rats was even higher than in DYOG (Figures 3A and 3B).

### Oxidative stress biomarkers and antioxidant parameters

As observed in Table 3, the oxidative stress was well-characterized in liver and plasma of STZ-diabetic rats, since the levels of MDA (liver or plasma) and PCO (liver) were increased in DYOG rats, and the activities of hepatic antioxidant enzymes SOD, CAT and GSH-Px were decreased, as well as the GSH levels. As expected, the treatment with insulin prevented the oxidative stress installation in liver of diabetic rats.

**Table 3:** Oxidative stress biomarkers (plasma and liver) and antioxidant enzymes activities (liver) of STZ-diabetic rats treated for 45 days with yoghurt enriched with curcumin, piperine, and curcumin+piperine.

Parameters	DYOG	DINS	DC90	DP20	DC90P20	DP40	DC90P40
Plasma MDA(µmol/L)	20.75±0.60	12.48±0.26(a)	12.57±1.35(a)	16.12±0.94(a)	12.44±0.97(a)	17.80±1.07(a, b, c, e)	19.42±1.46(b, c, d, e)
Liver MDA(µmol/L/g tissue)	198.69±4.26	115.37±6.49(a)	121.24±3.80(a)	128.24±5.63(a)	124.04±4.06(a)	135.23±6.55(a)	175.77±11.86(b, c)
Liver PCO(nmol/mg protein)	0.33±0.02	0.09±0.01(a)	0.15±0.02(a, b)	0.15±0.02(a, b)	0.16±0.02(a, b)	0.24±0.01(a, b, c, d)	0.26±0.01(a, b, c, d, e)
SOD(U/mg protein)	21.61±1.48	240.06±22.19(a)	191.72±11.23(a, b)	89.45±8.38(a, b, c)	114.69±8.42(a, b, c)	47.31±5.28(a, b, c, d, e)	33.37±5.29(b, c, d, e)
CAT(µKat/mg protein)	3.72±0.28	7.69±0.44(a)	6.05±0.24(a, b)	4.85±0.25(a, b, c)	5.91±0.36(a, b, d)	4.76±0.12(a, b, c, e)	4.40±0.31(b, c, e)
GSH-Px(µKat/mg protein)	0.52±0.04	1.01±0.07(a)	0.84±0.05(a)	0.81±0.04(a)	0.65±0.05(b)	0.57±0.08(b, c, d)	0.49±0.02(b, c, d)
GSH(mmol/L/g tissue)	20.54±2.24	52.93±1.67(a)	39.80±2.95(a, b)	34.48±3.95(a, b)	27.56±3.04(b, c)	27.66±2.65(b, c)	27.32±3.18(b, c)

Values are expressed as means ± SEM, n = 10. Differences between groups were analyzed with One-way ANOVA followed by Student-Newman-Keuls test. a, differences with DYOG; b, differences with DINS; c, differences with DC90; d, differences with DP20; e, differences with DC90P20 (p<0.05). Differences in a same group relative to day 0 were analyzed with Paired Student’s t test. #, differences with day 0 (p<0.05).

Although it has not achieved the magnitude of the insulin responses, the treatment of diabetic rats with curcumin-enriched yoghurt reduced the oxidative stress, since the levels of MDA and PCO were lower and the activities of antioxidant enzymes and the GSH levels were higher than those found in DYOG. Both curcumin +20 mg/kg piperine (DC90P20) promoted similar changes in the oxidative stress biomarkers and in CAT activity as those found in DC90; however, the antioxidant gluthatione system seems to be impaired in DC90P20 group, since both the GSH-Px activity and the GSH levels were similar those found in DYOG. SOD activity was increased in DC90P20 group when compared with DYOG, but was minor in comparison with DC90 (Table 3).

Decreased levels of MDA and PCO and increased activities of SOD and CAT were observed in rats of DP20 group and, in a lesser extent, in DP40 group. GSH-Px activity and GSH levels were increased in DP20 group, while these parameters were impaired in DP40 group, reaching values similar those found in DYOG. All markers of oxidative stress and antioxidant activities were similar between DYOG and DC90P40 groups, with exception of PCO (Table 3), denoting the absence of beneficial effects of the co-administration of curcumin +40 mg/kg piperine (DC90P40) against the oxidative stress in diabetes.

## Discussion

Data from this study reiterated previous findings of our laboratory [16], showing the antidiabetic activity of curcumin-enriched yoghurt in STZ-diabetic rats. Decreased levels of biomarkers of oxidative stress and of liver and kidney dysfunction were also observed. However, these curcumin effects remained lower than those of insulin, which could be a consequence of its low bioavailability. Attempting to improve the antidiabetic activity of curcumin, the present study investigated the changes in biochemical and physiological parameters of STZ-diabetic rats treated with yoghurt enriched with curcumin and/or piperine, since piperine has bioenhancer activity. Surprisingly, the treatment of diabetic rats with both curcumin and piperine did not bring any advantage to the curcumin effects; on the contrary, the co-administration with a higher dose of piperine impaired the curcumin benefits, suggesting that the biotransformation of curcumin is very important to its antidiabetic and antioxidant activities.

Various mechanisms have been recently described to explain the antidiabetic activity of curcumin. Stimulation of insulin release from pancreatic beta cells [41] could explain the various benefits observed in diabetic rats treated with curcumin-enriched yoghurt, such as reduction in glycemia (Figure 1) and subsequently glycosuria (Table 2), increase in the weight of adipose and muscle tissues (Table 1) and in the body weight gain (Figure 2) and reduction in the food intake (Table 2) in comparison with rats treated only with yoghurt. Increase in the skeletal muscle glucose uptake [42] and inhibition of hepatic gluconeogenesis [43] are also related with the antihyperglycemic effect of curcumin; both these mechanisms are associated with the ability of curcumin to activate the adenosine monophosphate-dependent kinase, AMPK. Activation of AMPK is also participating in the hypolipidemic effects of curcumin, which has been involved in the stimulation of fatty acid oxidation (increasing the expression of carnitine palmitoyl transferase-1) and inhibition of lipogenesis (decreasing the expression of glycerol-3-phosphate acyltransferase-1) in adipose tissue of obese mice treated with curcumin [44]; these data are in agreement with our findings about the reduction in the dyslipidemia of diabetic rats treated with curcumin-enriched yoghurt, which did not differ from those observed with the insulin treatment (Table 2).

Correction of blood glucose levels is an efficient mechanism to prevent the long-term complications of DM, mainly those related to oxidative stress and tissue damage. Increased liver exposure to ROS is associated with the onset of steatosis and its progression to steatohepatitis [45] and with various redox alterations that impair the activity of components belonging to signal transduction pathways [46]. According our data, oxidative stress in STZ-diabetic rats was well characterized, since we found high levels of LPO (MDA) and protein oxidation (PCO) biomarkers. Furthermore, the activities of antioxidant enzymes, as well as the levels of GSH, were decreased in the liver of STZ-diabetic rats (Table 3), as previously reported [47]. Low activities of antioxidant enzymes have been attributed to structural modifications promoted by both oxidative (due to increased O_2_^•−^ levels) and glycation processes [48]–[50]. The low levels of GSH in liver of diabetic rats may be a consequence of the increased glucose flux through the polyol pathway, consuming NADPH in the sorbitol formation, impairing the regeneration of GSH [51]. Taken together, the harmful impacts of oxidative stress in liver of diabetic rats may be noted by the progressive increase in the circulating levels of ALT, AST and ALP (Figure 3). Progressive elevation of these enzymes in plasma of diabetic rats was previously described [16], [40] and has a positive correlation with the increased release from tissues damaged by oxidative stress [52]. It is important to note that all these changes related to oxidative stress in diabetes were prevented by insulin treatment.

The treatment with curcumin-enriched yoghurt partially reversed the changes associated with oxidative stress in diabetic rats, increasing the activity of antioxidant enzymes and reducing the levels of oxidative stress biomarkers (Table 3). Studies have shown that curcumin possesses a potent *in vivo* antioxidant activity related to its ability to scavenge ROS [53], [54] and to inhibit LPO [55]. Therefore, both the ROS scavenger and the antihyperglycemic effect may be responsible for the *in vivo* antioxidant activity of curcumin. The increased GSH levels in liver of diabetic rats treated with curcumin can be also related to its antihyperglycemic effect, since less glucose undergoes the polyol pathway. Consequently, the increased GSH disposal may explains the increase in the GSH-Px activity in curcumin-treated rats. The restoration of the antioxidant system is helpful to combat the deleterious changes caused by ROS, leading to a fall in the MDA and PCO. Finally, the reduction in the hepatic oxidative stress by curcumin was corroborated by the diminished plasma levels of ALT, AST and ALP (Figure 3), in agreement with previous findings [16]. Curcumin-enriched yoghurt also promoted reduction in proteinuria (Table 2), which could be related to its effects against oxidative stress in kidney [56], [57].

It is well known that piperine increases the bioavailability of drugs by inhibiting various enzymes that participate in the hepatic and intestinal biotransformation and efflux of drugs. Although some studies had shown increase in curcumin bioavailability, the pharmacodynamic changes of this pigment when co-administered with piperine have been poorly studied. To our surprise, when diabetic rats were treated with both curcumin and piperine, no changes (curcumin:piperine, 90∶20) or even loss (curcumin:piperine, 90∶40) in the antidiabetic and antioxidant activities of curcumin were observed. Curcumin, when orally administered, undergoes rapid reduction and conjugation pathways occurring in the intestine and liver of human subjects [58] and rodents [59], [60]; these processes produce various curcumin metabolites, including dihydrocurcumin, tetrahydrocurcumin, hexahydrocurcumin, octahydrocurcumin, curcumin glucuronides and curcumin sulfate [19]. Therefore, increased circulating levels of curcumin are expected in the presence of piperine. Considering that the curcumin effects were not improved and they were even abolished in the presence of piperine, an increase in the circulating levels of curcumin may not be a useful approach to improve its pharmacodynamic actions, at least those related to antidiabetic and antioxidant activities. It is interesting to note that changes in the ALT, ALP (Figure 3), and GSH (Table 3) levels and in the GSH-Px activity (Table 3) were early detected in diabetic rats treated with curcumin +20 mg/kg piperine, showing that these biomarkers are more sensitive in monitoring the impairments of this association in diabetes. The complete absence of the curcumin effects when co-administered with 40 mg/kg piperine may be also related with the reach of toxic curcumin levels. Accumulating evidence has pointing that curcumin may have *in vivo* adverse effects when administered at very long periods [61] or under specific pathological conditions [62]. If a higher dose of piperine causes a major inhibition in the curcumin biotransformation, the probability of curcumin in reaching toxic levels at various tissues will be increased. The co-administration of curcumin and 40 mg/kg piperine led to a very increase in the ALT levels when compared with values of untreated diabetic rats (Figure 3), showing the toxicity of this co-administration in diabetes. The biological activities of curcumin metabolites have been a matter of extensive studies, alerting us for the caution when comparing the biological effects of curcumin evaluated through *in vivo*, *ex vivo* or *in vitro* approaches [18]. Studies demonstrated that tetrahydrocurcumin has antidiabetic and antioxidant activities greater than those of curcumin [63], [64]. Tetrahydrocurcumin also reduces the biomarkers of oxidative stress in liver and kidney [64], [65]. Recently, Neyrinck et al. [66] observed that mice fed a high-fat (HF) diet receiving curcuma extract (0.1% of curcumin) and 0.01% of white pepper (which contains piperine) for four weeks, showed increase in the body weight and fat accumulation, as well as impairments in glucose and lipid homeostasis, similarly to HF mice that did not receive the treatment, although the supplementation with these phytotherapics diminished the adipose tissue levels of the proinflammatory cytokines IL-6 and TNF-α. The authors did not study the effects of the phytotherapics in HF mice separately, so it cannot be affirmed that white pepper blocked the curcumin responses. However, this is a clear evidence that the co-administration of curcumin and piperine did not bring further benefits to the curcumin effects, at least in this situation of metabolic disorder related to obesity, corroborating our findings. It must be highlighted that the authors found tetrahydrocurcumin accumulation in the subcutaneous adipose tissue of HF mice receiving these two phytotherapics, and they suggested that the metabolite may be responsible for the down-regulation of proinflammatory cytokines.

Another unexpected finding of this study was related to the antidiabetic activity of 20 mg/kg piperine. However, no additional effects were observed when this minor piperine dose was co-administered with curcumin to diabetic rats. It is important to note that these two phytotherapics activate common intracellular effectors to control the metabolism of carbohydrates and lipids, such as AMPK, which could explain the absence of synergism when they are co-administered. In a study by Choi et al. [67], mice fed a HF diet and treated with piperine showed improvement in lipid profile, related to an increase in the plasma levels of adiponectin, which in turns stimulated the hepatic fatty acid oxidation and inhibited the lipogenesis in an AMPK-dependent mechanism. In the same study, it was also observed that piperine increased the phosphorylation of AKT in liver, and the authors associated the AKT activation with the increased GLUT2 translocation to plasma membrane of hepatic cells in HF mice, improving the glucose utilization. However, considering the well-established role of insulin in inhibit both the GLUT2 expression and its translocation to plasma membrane of liver cells [68], the phytotherapics effects on GLUT2 trafficking in liver must be interpreted with caution. Also, activation of AMPK and the subsequent stimulation of both lipid oxidation and tissue glucose uptake were also demonstrated for curcumin [44] and tetrahydrocurcumin [69]. Finally, given that 40 mg/kg piperine did not show the same beneficial effects, it can be suggested that higher doses of piperine reached toxic levels. Piyachaturawat et al. [70] found various histopathological changes related to toxicity in mice that received one-single administration of piperine in doses varying of 100 to 500 mg/kg; the authors comment that the use of piperine for a long period of time may reach toxic levels.

In conclusion, the present study provided clear evidences for the absence of additional effects in the antidiabetic and antioxidant activities of curcumin when administered with piperine to STZ-diabetic rats. The inhibition of biotransformation processes increases the bioavailability of drugs, however this strategy will not necessarily increase the pharmacodynamic actions, indeed, it can be favoring the reach of undesirable or even toxic levels, depending on the ratio drug:bioenhancer. Finally, the blockade of the curcumin benefits when administered with a higher dose of piperine evidences that the biotransformation of curcumin is very important to its antidiabetic and antioxidant activities.
